# African‐American men and prostate cancer‐specific mortality: a competing risk analysis of a large institutional cohort, 1989–2015

**DOI:** 10.1002/cam4.1451

**Published:** 2018-03-30

**Authors:** Vonetta L. Williams, Shivanshu Awasthi, Angelina K. Fink, Julio M. Pow‐Sang, Jong Y. Park, Travis Gerke, Kosj Yamoah

**Affiliations:** ^1^ Collaborative Data Services Core Tampa Florida; ^2^ Department of Cancer Epidemiology H. Lee Moffitt Cancer Center & Research Institute Tampa Florida; ^3^ Department of Genitourinary Oncology H. Lee Moffitt Cancer Center & Research Institute Tampa Florida; ^4^ Department of Radiation Oncology H. Lee Moffitt Cancer Center & Research Institute Tampa Florida

**Keywords:** African‐American men, competing risk analysis, disparity, other cause mortality, prostate cancer, prostate cancer‐specific mortality

## Abstract

Significant racial disparities in prostate cancer (PCa) outcomes have been reported, with African‐American men (AAM) more likely to endure adverse oncologic outcomes. Despite efforts to dissipate racial disparities in PCa, a survival gap persists and it remains unclear to what extent this disparity can be explained by known clinicodemographic factors. In this study, we leveraged our large institutional database, spanning over 25 years, to investigate whether AAM continued to experience poor PCa outcomes and factors that may contribute to racial disparities in PCa. A total of 7307 patients diagnosed with PCa from 1989 through 2015 were included. Associations of race and clinicodemographic characteristics were analyzed using chi‐square for categorical and Mann–Whitney *U*‐test for continuous variables. Racial differences in prostate cancer outcomes were analyzed using competing risk analysis methods of Fine and Gray. Median follow‐up time was 106 months. There were 2304 deaths recorded, of which 432 resulted from PCa. AAM were more likely to be diagnosed at an earlier age (median 60 vs. 65 years, *P* = <0.001) and were more likely to have ≥1 comorbidities (13.6% vs. 7.5%, *P* < 0.001). In a multivariate competing risk model, adjusted for baseline covariates, AAM experienced significantly higher risk of PCSM compared to NHW men (HR, 1.62, 95% CI, 1.02–2.57, *P* = 0.03) NHW. Among men diagnosed at an older age (>60 years), racial differences in PCSM were more pronounced, with AAM experiencing higher rates of PCSM (HR, 2.05, 95% CI, 1.26–3.34, *P* = 0.003). After adjustment of clinicodemographic and potential risk factors, AAM continue to experience an increased risk of mortality from PCa, especially older AAM. Furthermore, AAM are more likely to be diagnosed at an early age and more likely to have higher comorbidity indices.

## Introduction

Adenocarcinoma of the prostate (PCa) is the most prevalent type of cancer among men, with median age at diagnosis of 66 years 1. In 2016, approximately 180,890 patients were diagnosed with PCa and 26,120 deaths from PCa were reported 2. Significant racial disparities in PCa have been widely reported 3, with African‐American men (AAM) sharing the disproportionate burden of the PCa and more likely to endure worse prognosis compared to non‐Hispanic White (NHW) men 4, 5, 6. The survival gap between AAM and White men has also been widening over the last decade, in the USA 7. Furthermore, the incidence of PCa also remains higher among AAM compared to NHW. Between 2008 and 2012, the incidence rate among AAM in the United States (U.S.) was reported as 208.7 per 100,000, almost two times higher compared to NHW men (123 per 100,000) 2. Inadequate access to healthcare services, late diagnosis, and socioeconomic status (SES) are some of the contributing factors to the disparities associated with PCa treatment, outcomes, and survival 7, 8. In addition, studies have demonstrated a differential presence of biological variants associated with PCa among AAM; predisposing them to high risk of aggressive PCa 9. Quality of care (QOC) received for patient diagnosed with PCa also differs between AAM and their white counterparts, where AAM are more likely to have lower QOC which can negatively impact the clinical outcome of their disease 10.

Notwithstanding the growing body of literature supporting the existence of racial disparities in PCa, some studies have failed to detect racial difference in PCa outcomes 11, 12, 13, 14, 15. In the prospective PIVOT trial, authors found no difference by race for the effect of PCa treatment on both PCa specific and all‐cause mortality 12. Similarly, in a study by Graham‐Steed et al. 14, the authors argued that significant racial differences in PCa‐specific mortality (PCSM) do not exist when patients have equal access to health care. To reach consensus, it is imperative to carefully analyze and control for the variables that are known to impact PCa outcomes in order to make meaningful inferences about the magnitude of the race effect on PCa outcomes. Additionally, given the high evidence of indolent PCa with favorable prognosis, PCa patients are more likely to die with PCa, rather than from PCa, as a result of other comorbidities 16. The presence of these comorbidities may act as competing risk for PCSM and require careful analytic attention when making inference on factors that associate with PCSM 17. Therefore, in this study, we investigate whether racial disparities in PCSM persist after adjustment for the known clinicodemographic risk factors and competing risk in large institutional PCa patients cohort spanning over 25 years. The study leverages detailed information on various risk factors and outcomes available through the Health Research Informatics (HRI) platform of Moffitt Cancer Center (MCC).

## Materials and Methods

### Data source and study population

**Figure 1:**
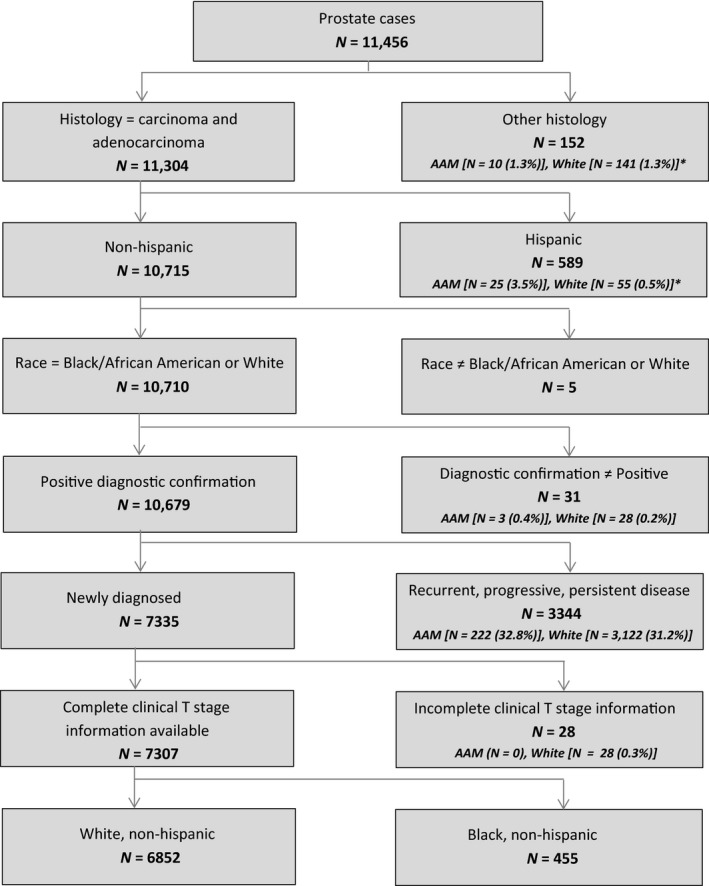
Consort diagram for the inclusion and exclusion criteria. *In consort diagram indicated the other race that were excluded in the analysis.

A total of 11,456 PCa patients were identified through the Health Records Informatics (HRI) platform at the Moffitt Cancer Center (MCC). The HRI platform is MCC's enterprise wide data warehouse which contains demographic, clinical, and outcome data that are collected from MCC data source systems. HRI includes data from MCC source systems such as the cancer registry, billing, bio‐banking, and the electronic medical record. PCa cases which did not meet the inclusion criteria, such as histology confirmation of adenocarcinoma, non‐Hispanic ethnicity, positive diagnostic confirmation, and complete information on clinical T stage, were excluded (*N* = 4149; Fig. 1). Based on the inclusion criteria, a cohort of 7307 men (94% NHW, 6% AAM), newly diagnosed with PCa at MCC between calendar year 1989 to 2015, was selected. Proportion of AAM and NHW excluded are highlighted in consort diagram (Fig. 1).

### Baseline covariates

Age at diagnosis, year of diagnosis, tobacco exposure, and health insurance status were used as primary demographic variables. Age at the time of diagnosis was categorized as <55, 55–64, 65–74, and >74 years of age. Tobacco exposure was categorized as never or ever based on patient self‐reported data captured at the time of initial diagnosis. Tobacco exposure was considered unknown if the exposure status could not be assessed or if it was not documented in the medical record. Health insurance can provide valuable information about the patient's ability to access essential healthcare services and thus can impact their disease control 18, 19. Additionally, health insurance status is also a good indicator for SES and can be used as a proxy for financial status of patients. In this study, health insurance status was determined based on the patient's primary payer at the time of initial PCa diagnosis and was categorized as private insurance, Medicare only (Medicare without supplemental coverage), Medicaid (Medicaid eligible patients), and uninsured 20, 21. Finally, the era of PCa diagnosis was categorized to account for the pre‐ and postprostate‐specific antigen (PSA) era, before calendar year (CY) 1993 versus after 1993 (inclusive) 22.

Comorbidity index, stage of cancer at presentation, and clinical T stage were used as clinical variables in the analysis. The Charlson‐Deyo comorbidity score was used to categorize the documented presence of comorbidities such as diabetes and heart disease in the patient record 23. The comorbidities were identified using the International Classification of Diseases (ICD)‐9th and 10th edition codes and were reported as 0 (no comorbidity documented in the medical record) or ≥1 (1 or more comorbidities documented in the medical record) 23, 24. Because studies have indicated higher likelihood of clinically advance PCa among AAM at the time of diagnosis 4, 25, a potentially critical source of confounding in the relationship between race and PCSM may be captured through T stage. Therefore, biopsy determined clinical T stage was used in the analysis to account for the clinical risk of the patients, that is, the extent of the disease before receiving any initial therapy and was categorized as T1–T2a, T2b–T2c, T3–T3a, and T3b–T4 26. Furthermore, in survival analysis, cases with distant metastasis (advanced PCa) at the time of presentation (*n* = 266) were excluded, mainly to limit the inclusion of only organ confined PCa cases and to balance the risk status in the multivariate model.

Information regarding patients' first course of treatment was identified and categorized for further comparison. First course of treatment was categorized as no treatment, surgery, radiation, and hormone therapy. The first course of treatment may have been administered as a single treatment (surgery only) or in combination with other treatment modalities (surgery and radiation). The unconventional treatment modalities such as chemotherapy and immunotherapy which were combined with radiation, surgery, and hormone therapy were categorized as radiation other, surgery other, and hormone other. In addition, patients who received unconventional therapy but their primary treatment information was not available were categorized as other therapy (*n* = 25).

### PCa outcome variables

To understand the disparities in survival outcome by race, PCSM and other‐cause mortality (OCM) were used as a primary endpoint. Survival time was calculated as the time from the date of diagnosis to date of last contact/death. PCSM was defined as death from PCa and was captured using cancer site codes from the International Classification of Diseases for Oncology, third edition (ICD‐O‐3 (C61.9), ICD‐9 (185) and ICD‐10 (C61) codes 24, 27. For PCSM, observations were considered censored if the death (events) were due to other documented causes or if the patient was alive by the end of follow‐up. For OCM, deaths due to causes other than PCa were considered as an event. Death was confirmed through multiple mechanisms, that is, for patients that died at the reporting facility, this information was documented in the electronic medical record and the patient vital status was subsequently updated in downstream databases. In addition, patient death was confirmed through a phone call from a spouse or relative or through death certificate data from state or national vital statistics.

### Statistical analysis

In this retrospective study, we aim to assess the extent to which race is an independent predictor of PCSM beyond known clinicodemographic risk factors. Associations between categorical variables and race were assessed via chi‐squared (χ^2^) or Fisher's exact test, and the Mann–Whitney *U*‐test was used to analyze the differences in continuous variables by race. Unadjusted Kaplan–Meier (KM) curves were used to assess 10‐year PCa survival within the strata of race. With long PCa survival time 16, death from other comorbidities (competing risk) is common 17. Therefore, the competing risk method of Fine and Gray was employed to estimate PCSM risk (Table 2). Briefly, the Fine and Gray method calculates the cumulative incidence of cause specific death and reports hazard ratios associated with increased mortality while controlling for covariates and accounting for other competing risks in the same model 28. To account for the event of interest in the competing risk model, PCa‐specific deaths were coded as 1, while death from non‐PCa or other causes were coded as 2. Patients who did not experience any event by the end of follow‐up were censored and coded as 0. Hazard ratios from the adjusted models were calculated using the eventcode function in the PHREG procedure as highlighted in SAS/STAT 13.1 29.

In addition, interactions between race and baseline covariates (age at diagnosis, treatment, tobacco exposure, health Insurance, clinical T stage, PSA era, and comorbidity Index) were also evaluated in multivariate survival model. In order to assess the independent association between race and survival outcomes, baseline covariates, that were differentially associated with race in categorical analysis with p value of <0.05 (Table 1), were also adjusted in the multivariate model. Furthermore, a sensitivity analysis was performed to evaluate the effect of unknown categories of clinical T stage and tobacco exposure variables on race and outcome association, by analyzing the multivariate models with or without unknown categories. All baseline covariates were considered categorical variables in the survival analysis. Hazard Ratio (HR), and 95% confidence intervals (CI) associated with outcomes were reported. SAS software version 9.4 (SAS Institute, Inc. Cary, North America) and R Statistical Software version 3.4.1 (R Foundation for Statistical Computing, Vienna, Austria) were used to perform all analyses.

**Table 1:** Baseline covariates

Clinicodemographic characteristics	AAM (*n* = 455) *N* (%)	NHW (*n* = 6852) *N* (%)	*P*‐value
Age at diagnosis (year*s*)			
Average (±SD)	60.4 ± 9.22	64.7 ± 8.67	<0.001
Median	60	65	
Inter quartile range	54–67	59–71	
Median follow‐up (months)	87	107	<0.001
Era of diagnosis			
Pre‐PSA era (<1993)	6 (1.3%)	216 (3.1%)	0.02
Post‐PSA era (≥1993)	449 (98.7%)	6636 (96.8%)	
Stage at presentation			
Localized	387 (85.0%)	5,822 (84.9%)	0.58
Regional	19 (4.1%)	344 (5.0%)	
Distant metastasis	21 (4.6%)	245 (3.5%)	
Unknown	28 (6.1%)	441 (6.4%)	
Clinical T stage			
T1–T2A	389 (85.5%)	5,237 (76.4%)	<0.001
T2B–T2C	37 (8.1%)	630 (9.2%)	
T3A	7 (1.5%)	135 (2.0%)	
T3B–T4	10 (2.2%)	204 (3.0%)	
Unknown	12 (2.6%)	646 (9.5%)	
Comorbidity (Charlson Comorbid Index)			
0	393 (86.37%)	6334 (92.44%)	<0.001
≥1	62 (13.63%)	518 (7.56%)	
Health insurance status			
Private insurance	306 (67.2)	4251 (62.0)	<0.001
Medicare alone	65 (14.3)	1667 (24.3)	
Medicaid coverage	24 (5.3)	67 (1.0)	
Uninsured	60 (13.2)	867 (12.6)	
Tobacco use			
Ever	219 (48.1%)	4239 (61.9%)	<0.001
Never	201 (44.2%)	2190 (31.9%)	
Unknown	35 (7.7%)	423 (6.2%)	
Treatment patterns^a^			
No treatment	49 (10.7%)	885 (12.9)	0.18
Radiation	181 (39.6%)	2381 (34.4%)	0.02
Surgery	195 (42.9%)	3123 (45.1%)	0.2
Hormone	28 (5.9%)	440 (6.3%)	0.8

AAM, African‐American men; NHW, non‐hispanic white men; PSA, prostate specific antigen.

a Does not include other unconventional treatment.

## Results

A total of 7307 histologically confirmed PCa patients were included in the study. Median follow‐up time of the study was 97 months. AAM were more likely to be diagnosed with PCa earlier in life compared to NHW men, with the median age at diagnosis 60 versus 65 years, respectively (*P* < 0.001). AAM were more likely to have Medicaid coverage compared to NHW men (5.3% vs. 1.0%, *P* < 0.001). NHW men were more likely to have tobacco exposure compared to AAM (61.9% vs. 48.1%, *P* < 0.001). AAM had a higher comorbidity index score compared to NHW men (13.6% vs. 7.5%, *P* < 0.001) (Table 1). Regarding first course of treatment, radiation and surgery were the primary treatment modalities compared to other unconventional therapies including hormone therapy. Furthermore, AAM were significantly more likely to receive radiation as first course of treatment compared to NHW men (39.6% vs. 34.4%, *P* = 0.02). There were no racial differences in treatment selection for those who did not receive definite treatment, those who underwent surgery (single or in combination with other treatment modalities) or those who selected hormone therapy (single or in combination with other treatment modalities) (Table 2).

**Table 2:** Multivariate competing risk Cox model to estimate the risk of prostate cancer‐specific mortality (PCSM) and other cause mortality (OCM).^a^

Parameters	No of deaths	Person months	Adjusted HR^b^ (PCSM)	*P* value	Adjusted HR^c^ (OCM)	*P* value
Race						
NHW	2020	759,541	*1 (Ref)*		*1 (Ref)*	
AAM	88	42,923	1.62 (1.05–2.57)	*0.03*	0.97 (0.76–1.25)	0.9
Age at diagnosis						
≤50	35	52,259	*1 (Ref)*		*1 (Ref)*	
>51–60	307	223,576	1.55 (0.70–3.45)	0.2	1.99 (1.33–2.97)	<0.001
>61–70	847	356,154	1.76 (0.80–3.87)	0.1	3.01 (2.03–4.44)	<0.001
>70	919	170,475	2.37 (1.05–5.34)	0.03	6.34 (4.26–9.43)	<0.001
Treatment						
No treatment	330	89173	*1 (Ref)*		*1 (Ref)*	
Radiation	2524	302,867	0.76 (0.52–1.10)	0.1	0.82 (0.71–0.94)	0.006
Surgery	739	378,583	0.63 (0.42–0.95)	0.02	0.63 (0.53–0.75)	<0.001
Hormone	190	30828	1.76 (1.12–2.75)	0.01	1.12 (0.90–1.39)	0.2
Clinical T stage						
T1–T2A	1336	629,025	*1 (Ref)*		*1 (Ref)*	
T2B–T2C	254	78,675	1.77 (1.25–2.52)	0.001	1.15 (0.99–1.33)	0.05
T3–T3A	66	13,447	4.86 (3.11–7.60)	<0.001	1.15 (0.83–1.60)	0.31
T3B–T4	115	18,546	5.54 (3.73–8.23)	<0.001	1.35 (1.06–1.73)	0.01
Unknown stage	337	62,771	2.20 (1.47–3.30)	<0.001	2.55 (2.12–3.07)	<0.001
Charlson Deyo comorbidity index						
0	2012	38,806	*1 (Ref)*		*1 (Ref)*	
≥1	96	763,658	0.75 (0.38–1.47)	0.4	1.31 (1.04–1.67)	0.02
Insurance status						
Uninsured	453	137,756	*1 (Ref)*		*1 (Ref)*	
Private insurance	798	448,801	0.84 (0.58–1.22)	0.3	1.0 (0.85–1.18)	0.9
Medicare alone	832	209,267	1.02 (0.70–1.49)	0.9	1.17 (1.00–1.37)	0.04
Medicaid coverage	25	6640	0.96 (0.28–3.25)	0.9	2.07 (1.32–3.25)	0.001
Tobacco exposure (self‐reported)						
Never	482	268,574	*1 (Ref)*		*1 (Ref)*	
Ever	1422	479,828	1.34 (1.01–1.77)	0.03	1.43 (1.28–1.60)	<0.001
Unknown	204	372	1.56 (1.04–2.34)	0.03	1.28 (1.06–1.54)	0.008
Era of diagnosis						
<1993	170	33,796	*1 (Ref)*		*1 (Ref)*	
≥1993	6839	768,668	0.41 (0.26–0.63)	<0.001	1.11 (0.88–1.39)	0.3

AAM, African‐American men; CI, confidence interval; HR, hazard ratio; NHW, non‐hispanic whites; PCSM, prostate cancer‐specific mortality.

a Model excludes the patients with distant metastasis at the time of diagnosis (*n* = 266).

b Adjusted Hazard ratio for the risk of prostate cancer‐specific mortality.

c Adjusted Hazard ratio for the risk of other cause mortality. Bold *p* value indicates the statistical significance for the Race difference in PCSM.

### Racial disparities in survival outcome

**Figure 2:**
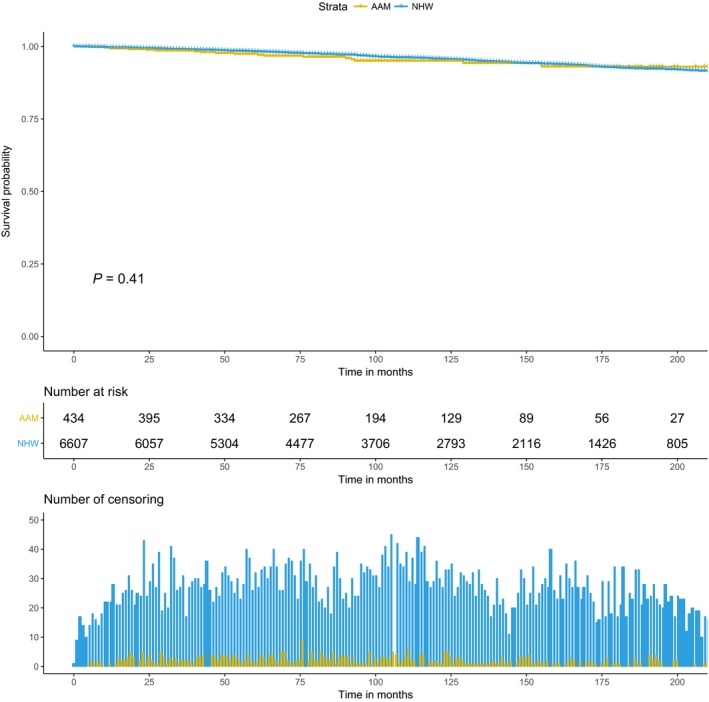
Kaplan–Meier curve for prostate cancer‐specific mortality within the strata of race.

There were a total of 432 documented PCa deaths and 1872 deaths due to other causes. In the competing risk analysis model (excluding cases with distant metastasis at the time of diagnosis), there were 307 deaths due to PCa. Unadjusted 10‐year PCa‐specific survival did not differ between AAM and NHW at 94% and 95%, respectively, log rank *P* = 0.4 (Fig. 2). However, in the multivariate competing risk model adjusted for important clinical and demographic variables, AAM had a significantly higher risk of PCSM (HR = 1.62, 95% CI, 1.02–2.57, *P* = 0.03) compared to their NHW counterparts. In a similar multivariate competing risk model looking at risk of OCM, a nonsignificant race effect was observed. Compared to NHW, AAM did not show any association with OCM, (HR = 0.97, 95% CI, 0.76–1.25, *P* = 0.9), Table 2.

### Baseline covariate and race disparities

**Figure 3:**
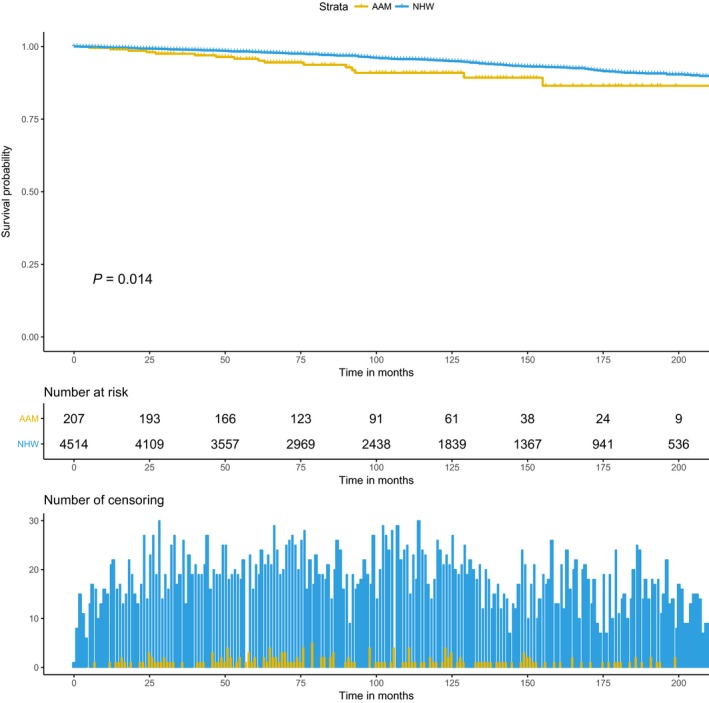
Kaplan–Meier curve for racial difference in prostate cancer‐specific mortality among men with age at diagnosis >60 years.

**Figure 4:**
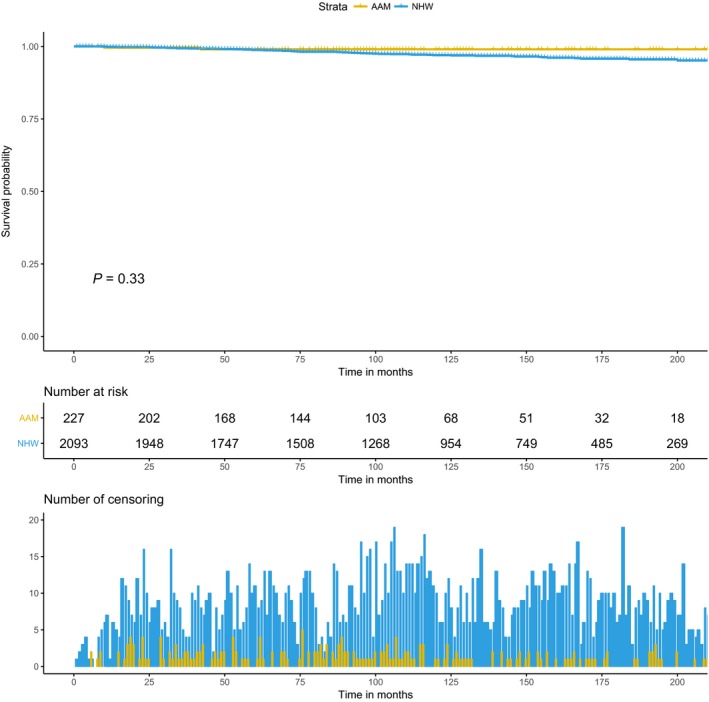
Kaplan–Meier curve for racial difference in prostate cancer‐specific mortality among men with age at diagnosis ≤60 years.

Baseline covariates which were differentially associated with race in categorical analysis were adjusted in the multivariate model. Furthermore, to assess the heterogeneity in race effect on PCa outcome in relation to baseline variables, the cross‐product interaction term between covariates and race was introduced in competing risk model. There was no interaction between race and other covariates for OCM outcome. For PCSM outcome, among all the covariates, age at diagnosis showed significant interaction with race (data not shown). Therefore additional age stratification was performed for the unadjusted KM analysis (Figs. 3 and 4), and the multivariate competing risk model (Table 3), for risk of PCSM by race. Age‐stratified KM curves reveal racial disparities in 10‐year PCSM among older patients (Figs. 3 and 4). In the age‐stratified multivariate model, for older patients (age at diagnosis >60 years), AAM were at significantly higher risk of PCSM compared to NHW (HR = 2.05, 95% CI, 1.26–3.34, *P* = 0.003) (Table 3). The age dependent race effect on PCSM was attenuated among the younger patients (age at diagnosis ≤60 years).

**Table 3:** Age stratified multivariate competing risk Cox model to estimate the risk of prostate cancer‐specific mortality within the strata of race

Race	Age at diagnosis ≤60 years		Age at diagnosis >60 years	
	Adjusted HR (95% CI)^a^	*P* value	Adjusted HR (95% CI)^a^	*P* value
NHW	*1 (Ref)*	–	*1 (Ref)*	–
AAM	0.71 (0.20–2.45)	0.5	2.05 (1.26–3.34)	**0.003**

AAM, African‐American men; CI, confidence interval; NHW, non‐hispanic white.

a Both multivariate Cox proportional hazard models are adjusted for Treatment, Clinical T Stage, Comorbidities, Smoking status, and PSA Era. Bold *p* Value indicates the statistical significance.

## Discussion

Several studies have shown that AAM are more likely to have aggressive PCa and are at higher risk of worse oncologic outcomes compared to NHW men 5, 6. In addition, AAM are at a higher risk of having adverse pathologic characteristics, biochemical recurrence, and worse overall survival after receiving PCa‐specific treatment compared to NHW men 25. The primary reason in the survival difference between races has been largely attributed to various socioeconomic and sociocultural factors 8. However, biological variability between AAM and NHW has also been identified as a major contributing factor in the disparities in disease characteristics and survival 9. This study aimed to explore whether AAM continued to have a higher risk of death from PCa even after adjusting for multiple risk factors. With the availability of substantial clinical and demographic data on various risk factors and a large study of AAM, we were able to make a robust assessment regarding the race effect on PCa outcomes.

This study demonstrates significant race differences in PCa outcomes among the large cohort of newly diagnosed PCa patients from a single tertiary cancer center. In our analysis, after adjustment for the potential risk factors and clinically relevant variables, AAM continue to experience higher PCSM, compared to NHW men. Our study preserve the trend observed in various studies which have shown significant race disparities in PCa with AAM enduring the worst survival outcome 13, 17, 30, 31, 32, 33. Using a similar approach to account for competing risk, a recent study by Wang et al. 17 reported high risk of PCSM among AAM. In another study by Tyson et al., authors reported significantly worse survival among AAM compared to white men 30. However, in another large study, mortality differences were not observed between AAM and NHW who had equal access to health care 14. Similarly, in our study, we did not observe any survival difference between race and PCa outcomes in the unadjusted KM analysis (Fig. 2). We suspect that the attenuated association between race and PCa mortality can possibly be attributed to the lack of statistical model adjustment of potential risk factors. If not controlled, known risk factors for PCa, such as age at diagnosis, health insurance status, presence of comorbidities, and tobacco exposure, can significantly impact the association between race and PCa outcomes 34, 35, 36, 37. Therefore, accounting for the presence of these known risk factors along with important clinical variables in our model reveal a persistent association between PCa outcomes and race. We also performed a sensitivity analysis (data not shown) to evaluate the effect of unknown categories of T stage and Tobacco exposure on the adjusted competing risk model (Table 3). Exclusion of unknown categories from the model did not distort the association between race and PCSM.

In addition to evaluating the race effect on PCa outcomes, we also explored the interaction between baseline covariates and race category in a multivariate competing risk model. Among all the covariates, age at diagnosis showed a significant interaction with race in PCSM model. Age at diagnosis plays an important role in PCa treatment as older age is associated with multiple competing risk factors and comorbid conditions that might influence treatment recommendations, and often associated with poor outcomes 34. In our multivariate age stratified model (Table 3), older AAM men (age at diagnosis ≥60 years) showed increased risk of PCSM compared to older NHW. Consistently, Du Xi and colleagues previously reported a marginally higher risk of PCSM among elderly AAM compared to NHW 38. Similarly, another study by Gornick et al. 39, authors demonstrated higher risk of mortality among older AAM and reported significant race difference in access to essential services among older patients. Consistent with other reports, our results also showed that AAM were more likely to be diagnosed at an earlier age compared to NHW men 15, 40, 41. In addition, a large proportion of AAM had higher comorbidity indices (Table 1). Studies have shown an increased likelihood of poor oncologic outcomes associated with higher comorbidities 42, especially among older patients 34, 43. Therefore, both early diagnosis and higher comorbidity may explain potentially higher disease burden and poor PCa prognosis among AAM 44. In the era of shared decision making in the selection of primary treatment of PCa, it is imperative to analyze race differences in treatment patterns. Consistent with previous studies 15, 45, we observed significant differences in the use of radiation therapy, with AAM more likely to receive radiation as primary treatment compared to NHW men. Moses and colleagues reported a similar pattern in their study with higher percentages of AAM receiving radiation over surgery 11, 15.

The major strength of this study is the availability of large clinical, demographic, and long term follow‐up data on PCa patients from a single tertiary cancer center; over 25 years of follow‐up data were included in this study. PCa is commonly a slow growing cancer, and deaths due to other competing risks are more common than death due to PCa itself 16, 46. Therefore, the long follow‐up in this study facilitated our ability to analyze the race effect on PCa‐specific outcomes. Furthermore, we also had detailed information on insurance status, tobacco exposure, comorbidities, and era of diagnosis that has not been traditionally adjusted for in a single large cohort of patients that specifically includes AAM patients. Therefore, we were able to adequately assess the influence of these variables on PCa outcomes. This study provides vital information on survival differences by race and will add to the current body of knowledge regarding PCa characteristics, treatment patterns, and long‐term outcomes.

Several limitations were identified in the conduct of this study. In order to capture as much information as possible, all patients who were evaluated at MCC with histologically confirmed PCa were included, thus making our study cohort one of the largest cohort of newly diagnosed PCa patients from a single institute with data spanning over 25 years. However, this presented a logistical challenge for us to capture vital information for the patients that were diagnosed before calendar year 1998, since the data before this time were captured through paper medical records and not hard coded in the electronic medical record system. In addition, PSA, primary Gleason pattern and secondary Gleason pattern, and Gleason score were not captured and hard coded with the Collaborative Staging data elements until 2004. As a result, we did not include PSA and Gleason in our analysis because the information was either incomplete or missing for most of the older cases. Lack of information on important clinical variables like PSA and Gleason limited our ability to fully account for the extent and aggressiveness of the disease or use the National Cancer Comprehensive Network (NCCN) risk stratification in our analysis 6. Additionally given the retrospective nature of the study and source of data used (single center cohort), issues pertinent to unmeasured confounding along with the generalizability of our results remains plausible. Another potential limitation of the study was possible misclassification of clinical T stage. T stage upgrading happens frequently after patients receive initial treatment 47. As we only included biopsy determined T stage (to adjust for the baseline risk difference), there was no control on possible T Stage upgrading, and that could lead to misclassification of patients risk status, post‐treatment. Loss to follow‐up was another study limitation. For example, patients who completed all or part of their initial first course of treatment at MCC, and subsequently left the local catchment area or the state of Florida and never return to MCC for further follow‐up visits may be considered lost to follow‐up. However, our Cancer Registry employs multiple attempts at ensuring we have minimum analytic (newly diagnosed) patients lost to follow‐up per the Commission on Cancer, American College of Surgeons accreditation standards and annual data submissions.

In conclusion, our study shows significant racial disparities in PCa outcomes, after receiving definite treatment. Upon adjusting for competing risk and multiple risk factors, AAM continue to experience significantly elevated rates of PCSM compared to NHW. Racial differences in PCSM were even more prominent among older AAM. Racial differences were not found for mortality due to other causes. Our evidence shows that, AAM are often diagnosed at an early age and are more likely to endure higher comorbidities compared to NHW which could explain increased disease burden among them. Future studies are required to replicate the findings of this study among other ethnic minorities and at‐risk populations.

## Conflict of Interest

None declared.
